# Experimental Analysis of Pressure Sensor Membranes Intended for Vacuum Arc-Extinguishing Chambers in Medium-Voltage Switching Devices

**DOI:** 10.3390/ma18245682

**Published:** 2025-12-18

**Authors:** Paweł Węgierek, Damian Kostyła, Paweł Okal, Czesław Kozak

**Affiliations:** Faculty of Electrical Engineering and Computer Science, Lublin University of Technology, Nadbystrzycka 38A, 20-618 Lublin, Poland; p.wegierek@pollub.pl (P.W.); c.kozak@pollub.pl (C.K.)

**Keywords:** membrane technology, pressure monitoring, membrane sensor, stainless steel sensor

## Abstract

This article presents a comparison of empirical and simulation studies and the parameters declared by the membrane manufacturer. The analysis concludes that these values differ at each stage. Therefore, a numerical and simulation analysis of an optimal flat membrane was undertaken, which will successfully perform measurement functions across the full pressure range without causing inelastic deformations based on a membrane made of 316 L stainless steel with the following mechanical parameters: Young’s modulus $E=2\times 10^{11}\;Pa$, Poisson’s ratio ν=0.28, density $\rho =7980\;kg/m^{3}$, and yield strength 2.8 × 10^8^ Pa. A diaphragm with an outer diameter of 25.4 mm, an inner diameter of $2.22\times 10^{-4}\;m$, and a thickness of t = $5.08\times 10^{-5}\;m$ was designed for a pressure sensor in vacuum extinguishing chambers of medium-voltage devices, with a pressure difference Δp from 7 × 10^−4^ Pa to 1.013 × 10^5^ Pa. Finite element method (FEM) simulations in the COMSOL Multiphysics environment showed maximum von Mises reduced stresses 1.96 × 10^8^ Pa below the yield strength, confirming operation in the linear-elastic range. The central deflection, described analytically by the equation $y=\frac{3(1-\nu^{2})Pr^{4}}{16Et^{3}}$, increased fivefold with an increase in diameter to $3.81\times 10^{-2}\;m$ (active area A = 1.14 × 10^−3^ m^2^ compared to 5.07 × 10^−4^ m^2^), achieving a metrological sensitivity of 9.1 × 10^−10^ m/Pa. Experimental studies integrated with Bragg FBG and epoxy adhesive (E = 5 × 10^9^ Pa, tensile strength $4.2\times 10^{7}\;Pa$) revealed a significant deviation from the manufacturer’s catalog data (e.g., deflection of $2.0\times 10^{-5}\;m$ at $6.89\times 10^{2}\;Pa$), resulting from uneven bonding and a lack of coaxiality. Corrugated membranes with t = $2.0\times 10^{-5}\;m$ exceeded plasticity, while the optimized configuration of a smooth membrane with rounded adhesive edges ($R=1\times 10^{-4}\;m$) enabled precise pressure monitoring below $10^{-1}\;Pa$, despite technological restrictions on assembly and miniaturization.

## 1. Introduction

The modern economy demands dynamic development of the energy sector, encompassing the exploration of new energy sources and methods of utilization through enhanced production efficiency and integration of diverse sources, ultimately ensuring the safe and efficient delivery of energy to both industrial enterprises and households [1].

Vacuum switching apparatus for medium-voltage applications has gained widespread use in power systems due to its high reliability [2], compact dimensions, and minimal maintenance requirements [3]. Vacuum devices constitute a fundamental component of such apparatus, ensuring effective arc extinction in a vacuum environment. The importance of vacuum technology is increasing in the context of developing environmentally sustainable energy infrastructure, particularly as an alternative to greenhouse gases such as SF6 [4].

It has been observed that degradation of vacuum within the arc extinction chamber, specifically an increase in pressure above 1 Pa, may result in sustained electrical arcing after contact separation. This phenomenon serves as a potential source of disturbances that can destabilize power line operation.

The measurement of actual pressure within sealed vacuum chambers during operation presents a significant technical challenge. Traditional methods of direct pressure measurement are virtually impossible to implement in operational devices due to the risk of damage to the measurement system caused by electrical discharge. Because of this limitation, indirect measurement techniques have been developed based on the analysis of the electrical properties of the chamber.

The advancement of micro- and nanotechnologies has enabled the miniaturization of transducers based on flexible plates [5], which has substantially increased their presence in both commercial and scientific applications [6]. In these systems, the membrane performs a dual function: structural—as a mechanically compliant element, and transductive—converting a physical stimulus (pressure, sound, or ion concentration) into a measurable signal [7]. A membrane sensor is a type of transducer in which the key detection component is a thin partition that deforms under the influence of the stimulus and generates a quantifiable response [8,9].

Designs of pressure sensors that combine a pressure-load-bearing component—such as the membrane [10,11], Bourdon tubes [12], elastic bellows [13], and other elements that deform under pressure load—with an optical fiber serving as the sensing element in pressure detection systems have gained significant popularity.

Circular pressure sensors with membranes have been extensively investigated regarding their sensitivity and performance across various applications. Research efforts have focused on optimizing structural components, such as membrane thickness and diameter, to achieve enhanced precision and response speed [14]. Diverse analytical techniques, including stress analysis and finite element modeling, have been developed to predict sensor behavior and output characteristics [15]. Recent studies have introduced analytical methods for the efficient computation of key parameters, such as contact point pressure and pull-in voltage, thereby providing a computationally efficient alternative compared to finite element analysis [16]. Although circular membranes are generally considered more sensitive than their square counterparts for the same active area, certain studies emphasize the advantages of square membrane configurations in specific applications [16,17].

Membrane sensors constitute a pivotal category of sensing devices in modern electronics, employing flexible membranes to detect changes in pressure, force, or other physical quantities. This technology has gained substantial prominence due to its capacity for achieving high sensitivity, reliability, and seamless integration with measurement systems.

Membrane sensors play a significant role in the context of pressure measurement in closed-structure vacuum electrical power equipment [18]. A key parameter determining the operational viability of such devices within the power system is the pressure prevailing in the inter-contact space. In gas-insulated apparatus, pressure monitoring is crucial, as the vacuum maintains its excellent insulating properties up to a pressure level of 1 × 10^0^ Pa. When the pressure exceeds this value, the breakdown voltage of the insulation gap drops sharply, which compromises the insulation capacity of the system. This combination of factors—the diminished insulation strength and the inability to verify the presence of a safe insulation gap between the contacts due to the sealed construction of the device—leads to the destabilization of power line operation [19].

Consequently, to address the identified research gap, conceptual work was undertaken on a membrane pressure transducer. The primary design objective was to achieve the device’s capability for continuous, dynamic data acquisition across a wide pressure range from 1 × 10^−4^ Pa to 1 × 10^5^ Pa, with precise calibration in the high-vacuum region (1 × 10^−4^ Pa to 1 × 10^0^ Pa). This region is characterized by vacuum maintaining its optimal insulating properties, which are crucial for the operation of switching systems. The measurement system, designed accordingly, constitutes a functional solution for integration with vacuum interrupter chambers used in medium-voltage switching apparatus, thereby enabling real-time condition monitoring.

## 2. Materials and Methods

In the design work associated with the development of a probe head for measuring pressure loads, it was determined that the key functional component would be an elastic membrane coupled with a fiber-optic measurement sensor. Consequently, empirical verification of the correct operation of the developed sensor design was performed. The experimental tests were conducted at room temperature (20 °C) and humidity (55%). It should be noted that the tested membrane was placed in a removable vacuum chamber, where the pressure was changed in accordance with the test program.

### 2.1. Structure and Empirical Tests of the Membrane Sensor

**Figure 1:**
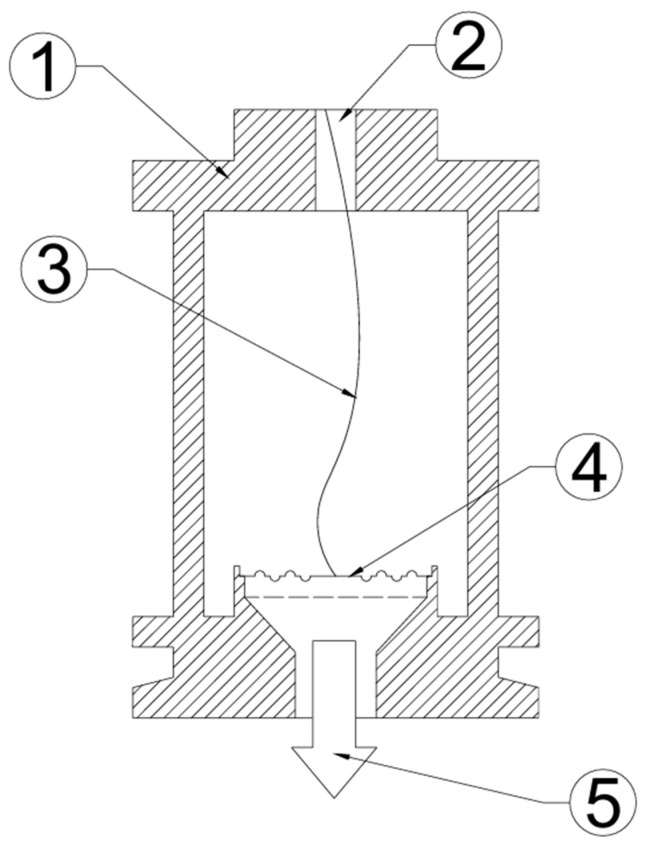
Structure of the pressure sensor. 1—Supporting structure; 2—guide element with a mount for optical fiber tensioning; 3—measurement optical fiber with a Bragg grating; 4—tested membrane; and 5—vacuum system with a vacuum pump.

Comparative tests and analyses were conducted on membranes manufactured from stainless steel and INCOTEL 718 alloy(Hudson Technologies, Ormond Beach, FL, USA), with a standard thickness of $2\times 10^{-3}\;m$ and a diameter of $2.54\times 10^{-2}\;m$. The membrane deflections for specified pressure values, as declared by the manufacturer, are presented in Table 1. The configuration of the measurement station required the design of a specialized mounting base, ensuring hermetic sealing and appropriate tensioning of the fiber Bragg grating (FBG). This design is illustrated in Figure 1.

The measurements required the bonding of the membrane to the optical fiber using a two-component epoxy adhesive. Within the optical fiber structure, a Bragg grating was inscribed—an area with a periodic modulation of the refractive index that acts as a narrowband optical filter, reflecting light at a specific wavelength known as the Bragg wavelength ($\lambda_{B}$). The integration of the individual sensor components enabled the formation of a complete signal processing chain. A change in pressure within the vacuum system caused deformation of the elastic membrane. The resulting variation in membrane deflection led to a change in the strain of the optical fiber containing the Bragg grating, which, in turn, produced a shift in the optical spectrum of the fiber Bragg grating, observed using an optical spectrum analyzer. The prevailing pressure in the system correlated with the spectral shift, enabling real-time detection of pressure variations within the setup.

The fiber Bragg grating used was manufactured using a phase mask method in an excimer laser system, with an irradiation wavelength of 248 nm, a phase mask period of 1068.97 nm, and a structure length of 12 mm. The grating had a reflection coefficient of approximately 0.5 and a very small full width at half maximum (FWHM) of 0.07 nm, which indicates a narrow and well-defined reflection peak. The spectrum is characterized by the absence of significant distortions and a fundamental maximum with a shape similar to a Gaussian distribution, with side modes of low amplitude (below 20%), which is important from the point of view of the high filtration and measurement quality of the grating.

**Figure 2:**
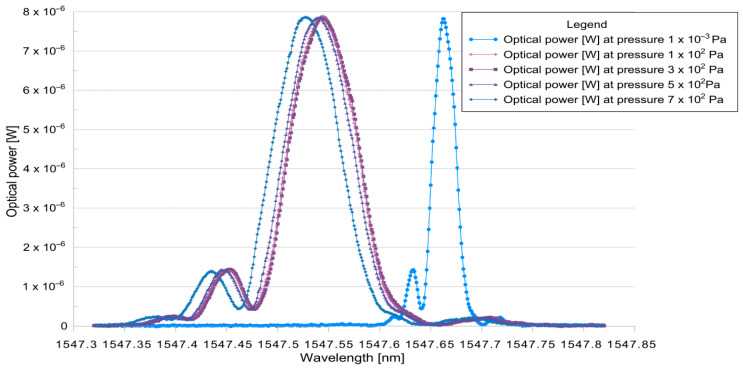
Empirical measurement results for a 316 stainless-steel membrane.

Empirical analysis of the relationship between membrane deformation and the applied pressure (Figure 2) revealed significant discrepancies in measurement accuracy and repeatability relative to the parameters declared by the manufacturer. The identification of technological constraints affecting the reliability of the results indicated that suboptimal adhesion during membrane assembly is a key factor limiting the precision of sensor positioning. In addition, challenges in maintaining the required component coaxially may have induced uneven pressure distribution (formation of gradients) on the membrane surface, leading to measurement errors. Another potential source of artifacts was the mounting of the fiber Bragg grating, whose presence could have interfered with the readings by inducing undesirable local stresses. Given the sources of interference, the obtained results should be interpreted as a preliminary basis for further analysis, subject to a limited capacity to reflect the actual mechanical characteristics of the examined membranes.

An empirical analysis of the relationship between membrane deformation and applied pressure (Figure 2) was performed by measuring the shift in the optical spectrum for individual pressures. This revealed significant discrepancies in the accuracy and repeatability of the measurements compared to the parameters declared by the manufacturer.

A series of empirical tests assessing the behavior of membranes under pressure loading revealed significant limitations of the experimental method when applied to complex mechanical systems. The interpretation of the recorded deformations was hindered by the constraints of the measurement apparatus and the influence of perturbing factors. The intricate geometry of the system and the multitude of parameters affecting membrane behaviors, such as stress nonuniformity, local material defects, and imperfections in boundary conditions, prevent both the extrapolation of the results and a comprehensive identification of potential structural critical zones. Furthermore, the laboratory conditions fail to fully replicate real loading scenarios, thereby restricting the scope of the acquired data.

The complexity of the mechanical system implies the insufficiency of empirical methods for a comprehensive characterization of membrane behavior. The identification of critical regions requires the analysis of stress and strain distributions, which is virtually infeasible by experimental means alone. Assessing the influence of geometric and material parameters would necessitate a costly and time-consuming series of tests across numerous variants, whereas predicting performance under extreme conditions—such as very high pressures or prolonged loading—requires modeling of processes that cannot be physically replicated in laboratory settings.

In view of the constraints, numerical simulations constitute an indispensable tool supporting the design and optimization of membranes. They enable high-resolution analysis of the spatial distribution of stresses and strains throughout the entire model volume, identification of potential locations for damage initiation, and effective optimization of geometric and material parameters through virtual testing of multiple structural variants. Furthermore, they allow for a reduction in research costs and time by minimizing the number of required empirical tests.

### 2.2. Assumptions of Numerical Analysis

Membrane pressure sensors require membranes characterized by a high sensitivity and linearity, meaning a proportional deformation to applied pressure, an appropriate elastic modulus ensuring mechanical strength, resistance to fatigue and overload, and thermal stability, expressed by low thermal expansion. Minimization of measurement errors necessitates reducing hysteresis to the lowest possible level. To achieve a fast dynamic response, the membrane mass must be minimized and its geometry must be precisely defined.

The materials employed must demonstrate long-term stability and reproducible properties. Particularly in metrological applications, the manufacturing process should enable cost-effective fabrication of thin structures and their integration with electronic components. For instance, silicon is used in small precision sensors, polymers are applied in medical systems, and ceramics are utilized in harsh or demanding environments.

To satisfy the requirements for the membrane, the stress range must remain below the material’s elastic limit. Therefore, the entire analysis can be conducted based on a linear elastic model. In such a model, a linearly elastic material is defined as one whose response to applied load remains elastic and obeys a linear relationship between stress and strain, up to the point where the elastic limit is exceeded.

(1) σ=E×ε

where

1. σ—stress in Pa,
2. E—modulus of elasticity (Young’s modulus) in Pa, which characterizes the stiffness of the material,
3. ε—relative deformation.

In such a material, the principle holds that deformations are reversible after the removal of the load, provided that the applied load has not exceeded the elastic strain limit.

In materials engineering and structural mechanics—especially in the design of thin-walled elements subjected to loads, such as membranes—numerical simulation of deformation constitutes an indispensable analytical and design tool. Conventional experimental methods, although essential for validation, are often costly, time-consuming, and constrained by the feasibility of testing under extreme conditions or with prototype geometries. Finite element method (FEM)-based simulations, on the other hand, offer the possibility of rapid, safe, and economical investigation of material behavior across a wide spectrum of loading and environmental conditions.

A key advantage of the simulation approach for metallic materials lies in its capacity for generalized modeling of various metal alloys using a relatively limited set of material parameters. To accurately capture the linear-elastic behavior of isotropic materials in FEM simulations, three fundamental properties are both necessary and sufficient.

Young’s modulus (E): The fundamental mechanical parameter defining the stiffness of a material—its ability to elastically deform under tensile or compressive stress. It is defined as the ratio of stress (σ) to strain (ε) within the elastic range of deformation.

(2) $$E=\frac{\delta }{\varepsilon }$$

The unit of Young’s modulus in the SI system is the Pascal (Pa), although in practice, gigapascals (GPa) or megapascals (MPa) are more commonly used. Young’s modulus (E) determines the magnitude of the displacements and deformations of a membrane under specified loads—higher values of E correspond to smaller deformations under the same loading conditions. Poisson’s ratio (ν) is a key parameter in the constitutive equations describing the stress–strain relationship. It affects the form of the stiffness matrix of elements. This dimensionless material parameter defines the ratio of transverse strain to longitudinal strain under uniaxial stress conditions. In finite element method (FEM) simulations, a higher ν value indicates greater lateral contraction during tension, which significantly influences the results of strength analyses. For isotropic materials, this parameter ranges from −1 to 0.5. Most metals exhibit a Poisson’s ratio of approximately ~0.3 (steel: 0.27–0.3); however, ν values close to 0.5 (for incompressible materials) require specialized computational techniques to avoid numerical errors. Mathematically, Poisson’s ratio is expressed by the following equation:

(3) $$v=\frac{\varepsilon_{transverse}}{\varepsilon_{elongated}}$$

where

ε_transverse_—deformation perpendicular to the applied force,
ε_elongated_—deformation in the direction of force action.

Material density (ρ) is a fundamental physical quantity that defines the mass per unit volume of a material, typically expressed in standard units $kg/m^{3}$. In the context of numerical modeling using the finite element method (FEM), its significance is particularly relevant in analyses that account for mass and the associated dynamic effects. Material density is essential for accurately representing mass and related physical phenomena in FEM models, and its correct specification is critical to the validity and reliability of engineering simulations.

Young’s modulus, Poisson’s ratio, and material density collectively define the fundamental elastic deformation capabilities of a metallic membrane within the elastic range. The fact that reliable modeling of various metallic materials in the elastic domain requires only the Young’s modulus, Poisson’s ratio, and density greatly simplifies the process of developing numerical models and highlights the essential role of these parameters in defining elastic mechanical and deformation characteristics, which are crucial for the performance of membrane elements. These parameters enable a quantitative description of the relationship between the applied load and the resulting deformation and geometric variation.

The use of these parameters makes it possible to develop a mathematical model from which the von Mises stresses can be determined. These stresses serve as a scalar measure of the equivalent or effective stress and are commonly used to assess the material strength of ductile materials (such as metals) under multiaxial loading conditions. They represent an equivalent normal stress that, in uniaxial tension, would produce the same energy effect as the actual complex stress state. For a triaxial stress system, the von Mises stress can be expressed by the following mathematical relation:

(4) $$\sigma_{v}=\sqrt{\frac{\left[{\left(\sigma_{1}-\sigma_{2}\right)}^{2}+{\left(\sigma_{2}-\sigma_{3}\right)}^{2}+{\left(\sigma_{3}-\sigma_{1}\right)}^{2}\right]}{2}}$$

where

$\sigma_{1}$, $\sigma_{2}$, $\sigma_{3}$—denote principal stresses.

For a flat system ($\sigma_{3}=0$), Equation (4) takes the following form:

(5) $$\sigma_{v}=\sqrt{\sigma_{1}^{2}+\sigma_{2}^{2}-\sigma_{1}\sigma_{2}}$$

The von Mises reduced stress (5) constitutes a sufficient criterion for analyzing the plastic deformation of ductile materials, particularly metals. Its theoretical foundations are based on the experimentally verified distortion energy theory, also known as the Huber–Mises–Hencky theory. This theory postulates that plastic yielding in a material begins when the energy associated with a change in shape (as opposed to a change in volume) reaches a critical value equivalent to the yield strength of the material determined from a uniaxial tensile test [21,22].

The sufficiency of the von Mises criterion arises from several fundamental properties. First, it enables the synthesis of an arbitrarily complex three-dimensional stress state into a single scalar measure. This scalar reduced stress is directly comparable to the uniaxial yield strength, allowing for effective prediction of plastic flow initiation at any point within a structure. Second, the criterion exhibits independence from the hydrostatic component of the stress tensor—that is, from stresses that act uniformly in all directions, either tensile or compressive. Therefore, it neglects the component that does not contribute to plastic yielding in metals, in accordance with experimental observations indicating that plastic deformations are primarily induced by shear stresses. Third, both common engineering practice and laboratory experiments empirically confirm that metal yielding occurs when the von Mises stress reaches the value of the uniaxial yield strength, irrespective of the loading condition—whether uniaxial, biaxial, or triaxial. Finally, in the context of numerical methods such as the finite element method (FEM), the use of von Mises stress significantly simplifies analysis. It provides a clear and automatic means to identify points in the model where the yield limit has been exceeded, thereby facilitating both the interpretation of results and the structural design process.

### 2.3. Numerical Analysis of the Empirically Tested Membrane

Membranes employed in pressure sensors are often intentionally designed with a corrugated (wavy) geometry, which significantly modifies their functional properties. The corrugated configuration increases compliance under applied pressure, allowing the detection of even minimal pressure variations, thereby enhancing the metrological precision of the device. Compared to smooth membranes of an equivalent thickness, the corrugated structure provides higher sensitivity within the low-pressure range. Additionally, the wave geometry promotes homogenization of stress distribution during deformation, reducing local stress concentrations. As a result, the membrane exhibits enhanced resistance to mechanical damage, extended service life, and the ability to operate over a wider pressure range.

The corrugated structure also minimizes material creep under prolonged static loading, ensuring improved shape recovery after unloading. Furthermore, it compensates for undesirable effects arising from differential thermal expansion, thereby limiting the influence of temperature variations on measurement accuracy. A key advantage lies in achieving optimal axial stiffness while maintaining high transverse flexibility—a critical parameter for precision applications.

**Figure 3:**
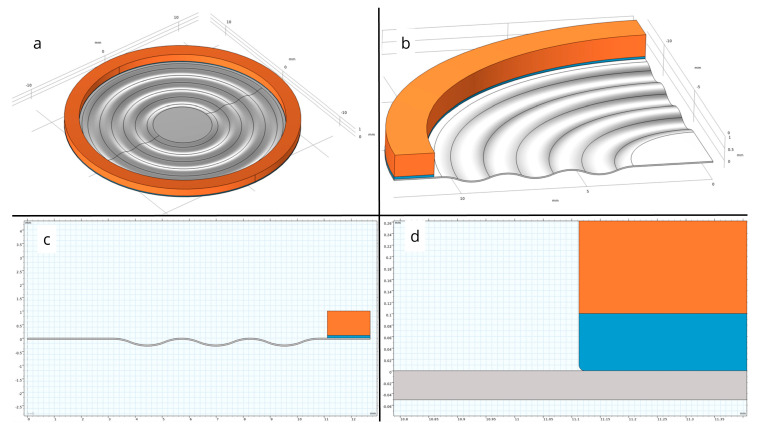
Structural details of the experimental object: (**a**) three-dimensional geometric model of the investigated membrane; (**b**) fragment of the cross-section of the simulation model; (**c**) cross-section of the modeled membrane, including the presence of adhesive; and (**d**) rounding of the adhesive edge to prevent numerical singularity. Orange color—measuring system mounting structure; Blue color—adhesive layer; Grey color—elastic membrane.

A numerical analysis was conducted in the COMSOL Multiphysics (software version 5.3) environment for a steel membrane with material parameters listed in Table 2. The axisymmetric nature of the problem enabled a significant reduction in computational complexity by restricting the simulation domain to a 2D model while preserving physical fidelity. In the developed model, which accurately represents the geometry of the actual component, the membrane was mounted onto a brass housing using an adhesive layer, with the properties specified in Table 2. The complete simulation model is illustrated in Figure 3a,b.

The finite element method (FEM) simulation allowed for detailed examination of the influence of geometric and material parameters on membrane behavior under specified pressure conditions. The model facilitated quantitative assessment of deformation resistance, analysis of structural stability, and verification of the permissible load ranges relative to the material’s strength limits. Additionally, the validation of the geometric configuration regarding the required load-bearing capacity formed the basis for evaluating the membrane’s suitability for target engineering applications. The region of the potentially highest stress was identified at the interface between the membrane and the adhesive edge. To prevent numerical singularity, the adhesive edge was rounded with a radius of 0.1 mm, as shown in Figure 3c,d.

The yield strength represents a key mechanical parameter of stainless steel type 316 (AISI 316) in the context of numerical modeling of material behavior, with its value of $2.8\times 10^{8}\;Pa$ listed in Table 2. The fundamental significance of this parameter arises from its definitional role in delineating the transition from the elastic to the plastic deformation domain under applied loads. In finite element simulations, the implementation of yield strength within the material’s constitutive model is mandatory due to its critical analytical function: it defines the upper boundary of the elastic range, indicating that operational stresses exceeding this threshold initiate permanent deformations; it enables prediction of plastic strains beyond the yield point, which is essential for assessing functionality under extreme loading conditions; and it provides the basis for computing safety factors by comparison with the maximum calculated stress values identified in the simulation. Omitting this parameter from the simulation setup would impose serious limitations on the validation of numerical results, precluding a reliable assessment of compliance with strength and operational criteria—an issue of particular importance in applications requiring full structural integrity under cyclic loading, such as precision membrane sensors operating under variable pressure conditions.

**Figure 4:**
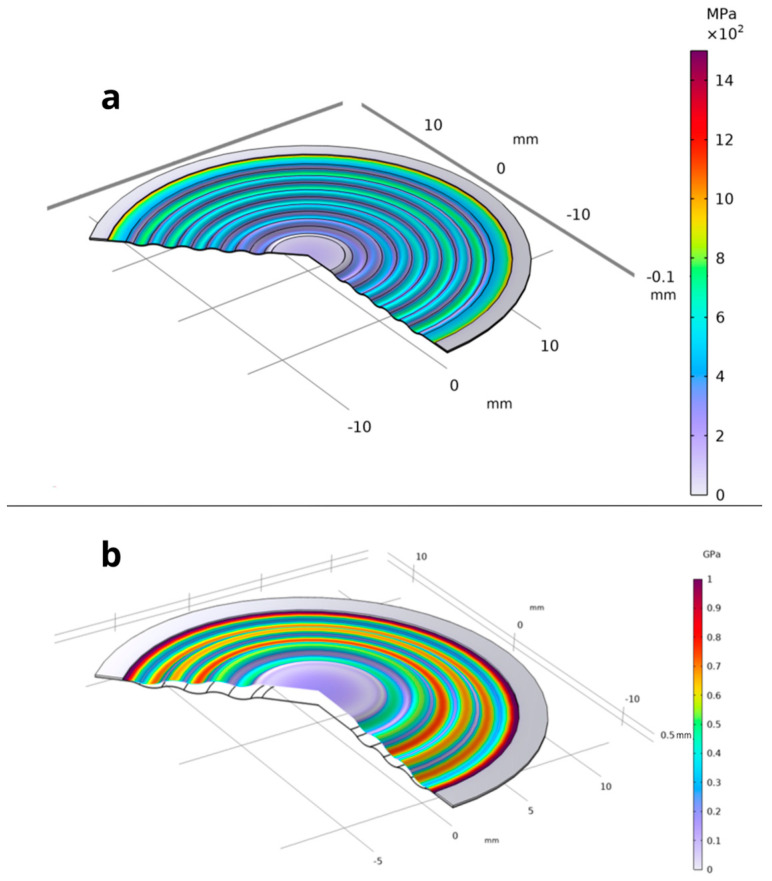
Distribution of von Mises stresses on the working plane along with the visible deformation—(**a**) membrane Ø 3.81 × 10^−2^ m and (**b**) Ø 2.54 × 10^−2^ m.

Considering the parameters presented in Table 2 and Table 3, the stainless-steel 316 membrane was subjected to a finite element analysis (FEA). Figure 4 illustrates a section of the analyzed membrane, showing the von Mises stress distribution and the deformation of the working surface.

**Figure 5:**
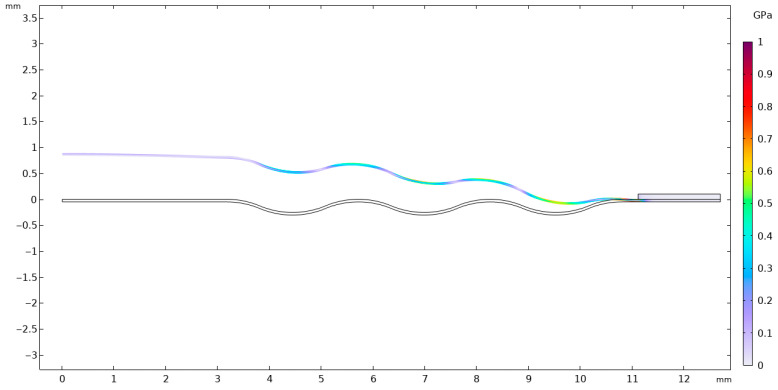
Von Mises stresses with corresponding 2D true strain.

**Figure 6:**
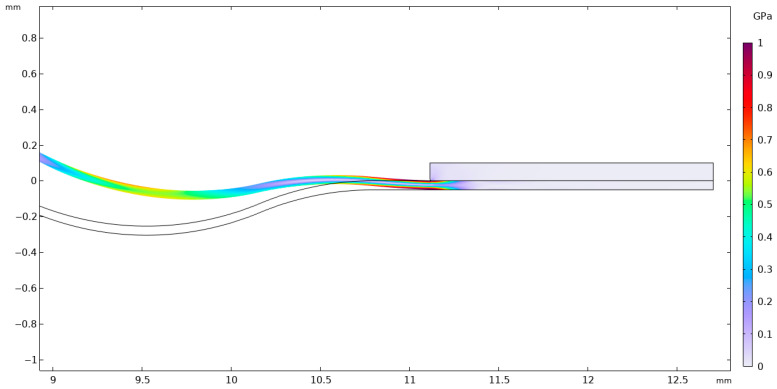
Von Mises stresses accompanied by the corresponding 2D true strain distribution in the region of maximum stress.

**Figure 7:**
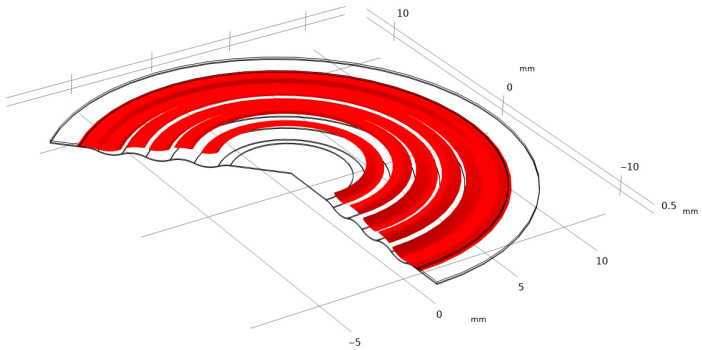
Von Mises stresses with the corresponding true strain shown above 2.8 × 10^8^ Pa in an isometric view.

**Figure 8:**
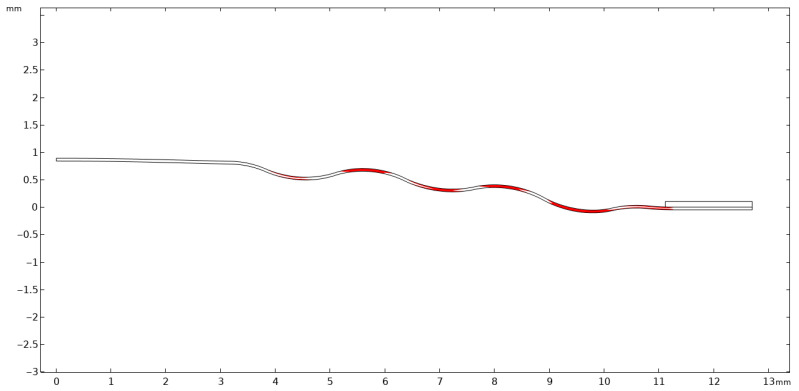
Von Mises stresses together with the corresponding 2D true strain in the section of stresses shown above 2.8 × 10^8^ Pa.

It is of fundamental importance to ensure that the analyzed stress–strain state remains within the linear-elastic range of the material, where Hooke’s law is valid. Exceeding the elastic limit—defined as the condition in which the von Mises equivalent stress surpasses the uniaxial yield strength of the material—indicates a departure from the range of applicability of the linear model. In such a case, further computational results cannot be regarded as quantitatively reliable for a conventional assessment of the elastic state, since the adopted model does not account for the material’s nonlinear behavior. Nevertheless, the occurrence and localization of elastic limit exceedances provide valuable engineering insights by revealing zones that are particularly susceptible to permanent deformation and forming a basis for more advanced analyses employing nonlinear material models. Figure 4, Figure 5, Figure 6, Figure 7 and Figure 8 demonstrate that Hooke’s law was not satisfied by the membrane manufactured from stainless steel 316.

The analysis of the examined membrane indicates that the material retains its elastic characteristics only under stress levels not exceeding $2.8\times 10^{8}\;Pa$. As shown in Figure 6 and Figure 7, the maximum von Mises equivalent stresses, particularly in the region of attachment to the housing, as well as in other membrane areas, exceed the material’s elastic limit. Considering that the yield strength of the material is $2.8\times 10^{8}\;Pa$, it can be unequivocally concluded that the elastic deformation range has been surpassed in multiple zones. Although the applied model does not allow for a quantitative assessment of the stresses in these regions, it enables the identification of critical areas and, therefore, provides sufficient grounds to conclude that the analyzed structure does not meet the requirements for elastic operation. Consequently, the obtained results clearly demonstrate that the employed membrane cannot be utilized under the specified loading conditions.

### 2.4. Numerical Modeling of a Membrane Meeting the Requirements of the Designed Sensor

The fundamental engineering challenge in designing membrane elements subjected to differential pressure (ΔP) lies in maximizing the achievable membrane deformation. Attaining the largest possible deflection is crucial for enhancing their functionality in high-sensitivity pressure sensors, where it directly affects operational parameters such as sensitivity, efficiency, and the measurement range of the detection system. This challenge arises from the necessity to achieve high mechanical compliance of the membrane, enabling significant elastic deformation under the applied pressure while simultaneously maintaining mechanical integrity—avoiding yield limit exceedance, structural failure, or undesired fatigue effects.

The mechanical stress of the modeled membrane is expressed by Formula (6).

(6) $$P=\frac{F}{A}$$

where F denotes the force acting upon the membrane, while A represents the area over which this force is applied. By transforming Equation (6), considering the membrane’s geometry and Newton’s Second Law of Motion, the above expression can be rewritten as follows:

(7) $$P=\frac{F}{A}=\frac{mg}{\pi r^{2}}$$

For a thin circular plate of radius r, with a material bending stiffness D, subjected to pressure loads P, the central deflection is described by Equation (8).

(8) $$y_{max}=\frac{{Pr}^{4}}{64D}$$

The parameter (D), representing the bending stiffness of the material, is described by Equation (9) [23,24].

(9) $$D=\frac{{Et}^{3}}{12\left(1-\vartheta^{2}\right)}$$

where E and t denote, respectively, the Young’s modulus and the thickness of the cross-sectional area of the analyzed membrane, while ϑ represents the Poisson’s ratio of the membrane material. By substituting Equation (9) into Expression (8) and simplifying the resulting formula, we obtain the following:

(10) $$y_{max}=\frac{{3\left(1-\vartheta^{2}\right)Pr}^{4}}{16{Et}^{3}}$$

where

y_max_—maximum deflection of the membrane center expressed in meters [m];
ϑ—Poisson Ratio;
P—pressure in the system expressed in Pascals [Pa];
E—Young’s modulus expressed in Pascals [Pa];
r—membrane radius expressed in meters [m];
t—membrane thickness expressed in meters [m];

The magnitude of the central deflection of a membrane is determined by the following three key parameters: the membrane diameter, its thickness, and the material’s Young’s modulus. The membrane radius plays a dominant role in generating large deformations. According to linear theory, the central deflection of a circular membrane rigidly clamped at its edges increases proportionally to the fourth power of the radius, which implies that even a small increase in this parameter results in a substantial amplification of the deflection [25]. In contrast, membrane thickness is a strongly limiting factor, as the deflection decreases proportionally to the third power of thickness [26]. Designing thinner membranes is, therefore, an effective means of enhancing deflections; however, this involves a significant trade-off: thinner structures are more susceptible to mechanical damage, and for deflections exceeding the membrane thickness, nonlinear phenomena become dominant. The Young’s modulus of the material, representing its stiffness, also constrains the deformation, since the deflection is inversely proportional to this parameter.

The consideration of geometric and material factors in the design process of measurement membranes necessitates a quantitative mathematical analysis, as only such a procedure enables a comprehensive understanding of the interdependence between diameter, thickness, and the elastic properties of the material. Although it is qualitatively known that a larger diameter leads to increased deflection, a thinner structure further amplifies it, and higher material stiffness constrains it, the actual influence of these parameters can be captured only through functional relationships.

### 2.5. Determination of the Technical Parameters of the Desired Membrane

Upon selecting the parameters of the elastic membrane intended for use in pressure sensors operating within the range from $1\times 10^{-4}\;\mathrm{Pa}\;$ to $1.013\times 10^{5}\;\mathrm{Pa}$, primary attention was directed toward determining the optimal technical characteristics of the membrane, taking into account dimensional and technological constraints associated with the target location of the measurement sensor installation.

(11) $$A=\pi r^{2}$$

(12) $$\frac{A_{2}=\pi r^{2}}{A_{1}=\pi r^{2}}=\frac{3.141593\times {\left(19.05\right)}^{2}}{3.141593\times {\left(12.07\right)}^{2}}=\frac{{1140.1\;mm}^{2}}{{506.7\;mm}^{2}}=2.25$$

where

- A_1_ − membrane ∅ = 2.54 × 10^−2^ m
- A_2_ − membrane ∅ = 3.81 × 10^−2^ m

Using the relationship expressed by Equation (12), membranes with diameters of $2.54\times 10^{-2}\;m$ (1”) and $3.81\times 10^{-2}\;m$ (1.5”) were analyzed as optimal in terms of available installation space. The membrane with a diameter of $2.54\times 10^{-2}\;m$ exhibited an active surface area of $5.067\times 10^{-1}{m\;}^{2}$, whereas the membrane with a diameter of $3.81\times 10^{-2}\;m$ provided an active surface area of $1.1401\times 10^{0}\;m^{2}$ [27,28,29]. This implies that selecting a membrane with a larger diameter increases the active surface area by $6.334\times 10^{-1}\;m^{2}$, corresponding to a 125% increase in the active surface area.

During mathematical modeling, to minimize the number of variables, a literature review determined that the typical Poisson’s ratio for metals ranges from 0.2 to 0.35 [30,31]. In engineering practice, Poisson’s ratio value of 0.3 is commonly accepted as the standard for most metallic materials. It is considered sufficiently accurate for the majority of strength calculations pertaining to structures made of steel, cast iron, and other metal alloys [31,32].

The maximum deformation of the membrane was also analyzed, which, according to Equation (10), increases proportionally to the fourth power of its radius ($r^{4}$). The membrane parameters adopted for comparative analysis are presented in Table 4.

- Material: Stainless steel 316,
- Young’s modulus: $2\times 10^{11}\;Pa$,
- Poisson’s ratio: 0.28,
- Applied pressure: $1.013\times 10^{3}\;Pa$,
- Thickness: $1\times 10^{-5}$ m,
- Yield strength: $2.8\times 10^{8}\;Pa$.

Assuming constant operating conditions and plates made of materials with identical physical properties, a more than fivefold increase in the deflection of the membrane of a given diameter is obtained, at $3.81\times 10^{-2}\;m$. This difference suggests a significant rise in deflection compliance with an increasing membrane diameter, which—assuming the model’s validity—could form the basis for considering the technological advantage of the larger variant in sensing applications. It is also worth noting that, from a technological standpoint, membranes with such diameters of $3.81\times 10^{-2}\;m$ are widely employed as low-pressure sensors [33].

Considering the results obtained above, a membrane with a diameter of Ø was adopted for further numerical analysis, Ø $3.81\times 10^{-2}\;m$.

The key aspect of the numerical analysis remains the behavior of the membrane within the range of elastic deformations, which necessitates that the following condition be satisfied:

(13) $$\sigma_{{max}_{\left(p_{max}\right)}}\leq \sigma_{gr}$$

(14) $$\sigma_{{max}_{\left(p_{max}\right)}}=\frac{3{P_{max}r}^{2}}{4t^{2}}$$

where

$\sigma_{{max}_{\left(p_{max}\right)}}$—maximum bending stress,
σ_gr_—elastic limit of a material.

According to the ASME Boiler and Pressure Vessel Code (BPVC), the coefficient of 0.67 serves as a conventional basis for determining the allowable stresses in materials used for pressure vessel construction. This implies that the calculated allowable stress shall not exceed the minimum yield strength of the material or—depending on thermal conditions and normative criteria—its ultimate tensile strength.

By substituting into Relation (13) the formula for the maximum bending stress at the central point (14) and considering the safety factor of 0.67, the expression assumes the following form (15):

(15) $$\frac{3{P_{max}r}^{2}}{4t^{2}}\leq 0.67\sigma_{gr}$$

By transforming Equation (14), it is possible to derive a relationship that determines the minimum thickness of the metal plate in relation to the maximum stress to which it will be subjected, as described by Equation (16).

(16) $$t\geq r\sqrt{\frac{{3P}_{max}R^{2}}{4\times 0.67\times \sigma_{gr}}}\;t\geq \sqrt{\frac{3\times 1.013\times 10^{5}\times {\left(1.905\times 10^{-2}\right)}^{2}}{4\times 0.67\times {(2.8\times 10}^{8})}}\;t\geq 4.684\times 10^{-4}\left[m\right]$$

By applying Relation (16) to the data provided in Table 4, it follows that the minimum thickness of the membrane must not be less than the specified value of $4.68\times 10^{-4}\;m$.

In addition to having the appropriate thickness, the membrane must also satisfy the condition of small deformations, as defined by Relation (17).

(17) $$t\geq \sqrt[{4}]{\frac{{3\left(1-\vartheta^{2}\right)Pr}^{4}}{0.2\times 16\times E}}\;t\geq \sqrt[{4}]{\frac{{3\times \left(1-0.28^{2}\right)\times (1.013\times 10^{5})\times (1.905\times 10^{-2})}^{4}}{0.2\times 16\times (2\times 10^{11})}}\;t\geq 4.997\times 10^{-4}\left[m\right]$$

Considering the specified minimum thicknesses of the metal plate resulting from the stress limitation condition (16) and the small strain condition (17), it should be assumed that the minimum material thickness shall not be less than $4.99\times 10^{-4}$ m. In practical terms, this allows one to conclude that the minimum thickness of the steel plate should be taken as no less than $5.00\times 10^{-4}$ m.

The obtained plate thickness $t=5.00\times 10^{-4}\;m$ and radius $r=1.905\times 10^{-2}\;m$ make it possible to classify the metal plate as a thin plate, since according to Relation (18), it satisfies the following condition $10\leq \frac{r}{t}\leq 100.$

(18) $$10\leq \frac{r}{t}\leq 100\;10\leq \frac{\left(1.905\times 10^{-2}\right)}{{(5\times 10}^{-4})}\leq 100\;\;10\leq 38.1\leq 100$$

The results obtained and the adopted assumptions indicate that the maximum deflection of the thin metal plate, as described by Equation (19), amounts to $9.221\times 10^{-3}$ m.

(19) $$y_{max}=\frac{{3\left(1-\vartheta^{2}\right)Pr}^{4}}{16{Et}^{3}}\;y_{max}=\frac{{3\times \left(1-0.28^{2}\right)\times 1.013\times 10^{5}\times (1.905\times 10^{-2})}^{4}}{16{\times (2\times 10^{11})\times {(5\times 10}^{-4})}^{3}}\;y_{max}=9.2213\times 10^{-5}\left[m\right]$$

The key simplifying assumption in the analysis is that the maximum deflection $\gamma_{max}$ remains significantly smaller than the plate thickness t. In engineering practice, the validity condition for the linear approximation is expressed as follows:

(20) $$\frac{\gamma_{max}}{t}\leq \;0.2\;\frac{9.2213\times 10^{-5}}{{5\times 10}^{-4}}=1.84\times 10^{-1}\leq \;2.0\times 10^{-1}$$

At higher values of this ratio, significant membrane effects become apparent—tensile stresses in the mid-plane that induce nonlinear shear coupling and necessitate a transition to nonlinear kinematics within the framework of large displacement theory.

Table 5 shows the linear response of the membrane to the applied pressure load.

(21) $$k=\frac{\gamma_{max}}{p}=\frac{\frac{r^{4}}{16Et^{3}}}{3\times \left(1-\vartheta^{2}\right)}\;k=\frac{{3\left(1-\vartheta^{2}\right)r}^{4}}{16{Et}^{3}}\;k=\frac{{3\times \left(1-0.28^{2}\right)\times (1.905\times 10^{-2})}^{4}}{16\times {\left(2\times 10^{11})\times {(5\times 10}^{-4}\right)}^{3}}\;k={9.103\times 10}^{-10}\;m/Pa$$

**Figure 9:**
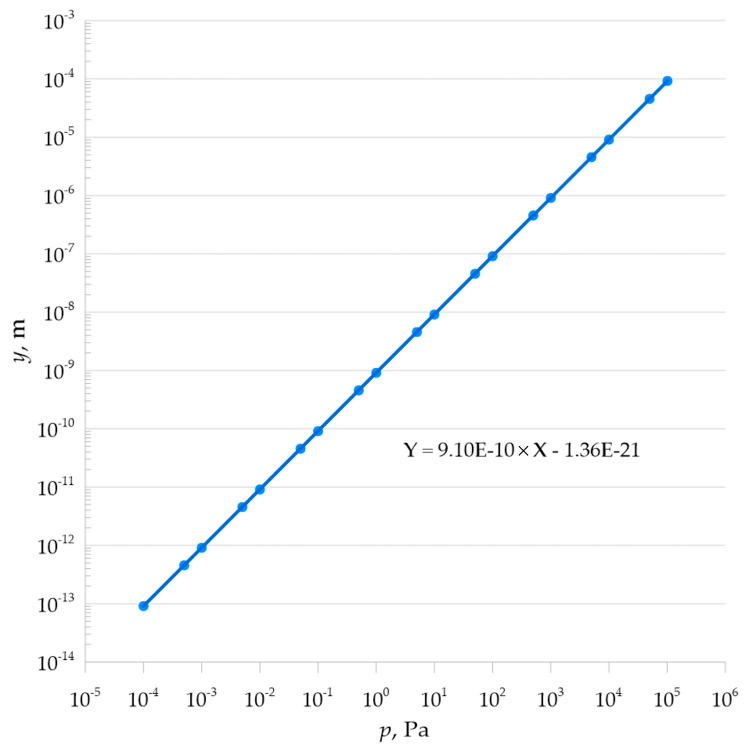
Performance characteristic of the metal plate expressed in meters per Pascal.

**Figure 10:**
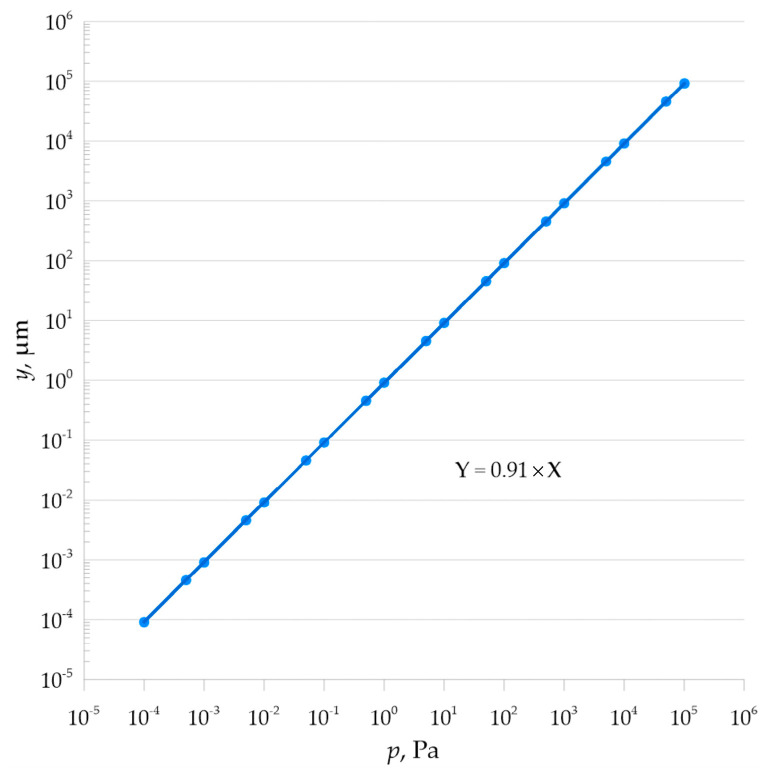
Operational characteristics of the metal plate expressed in nanometers per Pascal.

Mathematical analysis showed linear characteristics of the pressure load-bearing element, as shown in Figure 9 and Figure 10.

## 3. Research Results and Their Analysis

**Figure 11:**
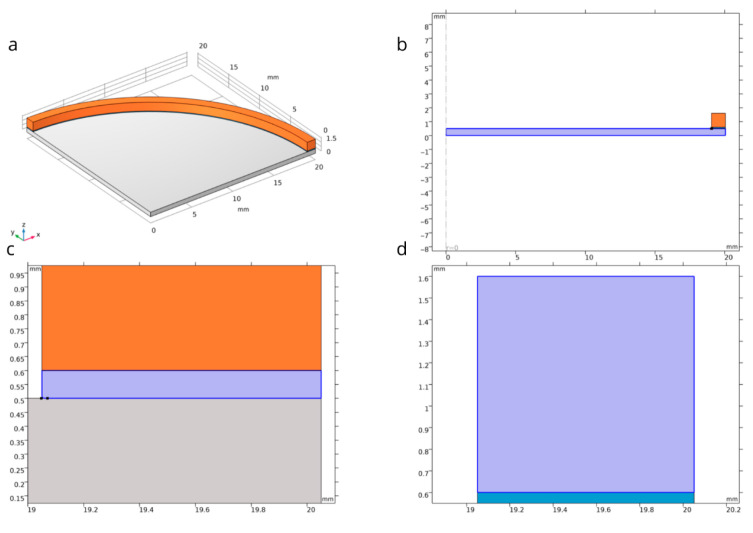
Structure of the simulation model: (**a**) geometric model of the analyzed component; (**b**) 2D view of the simulation setup; (**c**) rounding of the adhesive edge to prevent numerical singularity; and (**d**) contact zone between the adhesive layer and the vacuum vessel structure. Orange color—measuring system mounting structure; Purple color—adhesive layer; Grey color—elastic membrane; Blue color—vacuum vessel structure.

The geometric model illustrated in Figure 11 depicts a cross-section of the membrane together with the adhesive layer and the brass vessel. The dimensions are expressed in millimeters, enabling precise replication of the geometry. The analysis considers a pressure differential, where the external pressure is ${1\times 10}^{5}$ Pa and the internal pressure is ${1\times 10}^{-4}$ Pa, thereby simulating vacuum conditions. This loading induces deformations of the membrane and generates stress throughout the entire system.

The adhesive layer, which bonds the membrane to the brass vacuum chamber, constitutes a critical component of the structure. This intermediate layer serves as a mechanical buffer, distributing loads and dissipating forces that arise at the interface between two materials with differing mechanical properties. The mechanical characteristics of the adhesive play a decisive role in the stress distribution within the membrane and directly influence the overall mechanical behavior of the assembly underload. Analysis revealed that favorable adhesive properties enable effective redistribution of stress concentrations in the region where the membrane interfaces with the brass substrate. This prevents the formation of localized stress concentrations that could lead to structural damage such as cracking or irreversible deformation. Consequently, the adhesive layer functions as a mechanical stress damper, enhancing the structural integrity and durability of the membrane.

The numerical computations were performed in the COMSOL Multiphysics environment utilizing the Structural Mechanics module. Due to the geometric symmetry of the analyzed system and to reduce computational costs while maintaining an accurate representation of the investigated phenomenon, a two-dimensional axisymmetric model was employed. This approach allowed for precise mapping of the membrane’s stress and strain states while significantly reducing the number of degrees of freedom and facilitating the computational analysis process.

Discretization of the analyzed domain was conducted using an automatically generated adaptive mesh, whose density was dynamically modified based on the local gradients of selected physical quantities. As a result, in regions exhibiting the highest intensity of stress and displacement variations—particularly in areas critical to membrane deformation, the mesh density was considerably increased. The quality of the finite elements was verified through an analysis of the skewness parameter, which reached a value indicative of very favorable element geometry, providing the necessary condition for numerical stability and reliability of the computational results. The application of the adaptive meshing procedure additionally enabled a reduction in the total number of elements without compromising the required accuracy, thereby improving the overall computational efficiency of the simulation process.

The computations were carried out in the stationary analysis mode (Stationary Solver), accounting for geometric nonlinearity, which was resolved using the Newton–Raphson algorithm as the primary iterative method. The system of linear equations was solved through a direct method employing the MUMPS (MUltifrontal Massively Parallel sparse direct Solver) solver. Within this procedure, threshold pivoting with a value of 0.01 was implemented to ensure numerical stability when solving large and potentially ill-conditioned systems of equations.

The key element determining the credibility of the obtained results was the adoption of appropriate convergence criteria for the iterative process. In the conducted simulations, iterations continued until a relative tolerance of 0.001 was achieved, corresponding to the required reduction in the norm of the residual computational error, which quantifies the difference between the current approximation and the solution of the full set of nonlinear equations. Furthermore, the maximum number of iterations was limited to 25, enabling control of computational time in cases of non-convergence. The minimum damping factor was defined at ${1\times 10}^{-4}$ Pa, allowing the adjustment of step size in iterations under conditions that impeded the attainment of a stable solution. The Newton method was adopted as the termination criterion, implying that iterations were terminated exclusively upon fulfillment of convergence conditions associated with the Newton–Raphson algorithm. This configuration of solver parameters represents a compromise between computational efficiency and accuracy, while simultaneously minimizing the risk of premature process termination or the occurrence of unstable results.

A uniform load, represented by a constant pressure differential, was applied to the membrane surface. The analysis was restricted to static conditions, implying the omission of dynamic effects and the associated time-dependent nonlinearities. This simplification allowed the study to focus on the mechanical response of the membrane arising from the interaction between the pressure load and the geometric compliance of the system, in accordance with the static equilibrium equation and the displacement distribution.

(22) $$\nabla \cdot s+f_{v}=0$$

(23) $$u\left(R,\Phi ,Z\right)\to {\left(u,0,w\right)}^{T}$$

where

s—Cauchy stress tensor,
∇s—stress tensor divergence, i.e., internal forces,
fv—vector of volume forces
u—displacement vector in a cylindrical coordinate system (R, Φ, Z),

It is assumed that the system exhibits axial symmetry, implying the absence of displacements in the circumferential direction Φ. The function u(R,Z) denotes the displacement in the radial direction R, while w(R,Z) represents the displacement in the axial direction Z.

**Figure 12:**
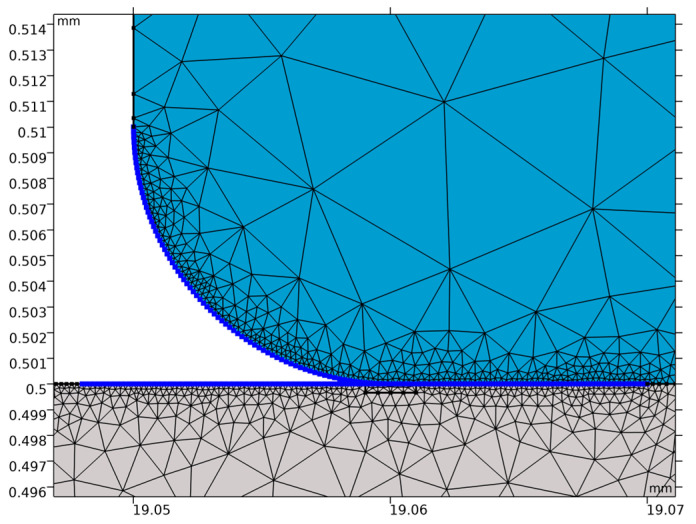
Adhesive–membrane interface region.

**Figure 13:**
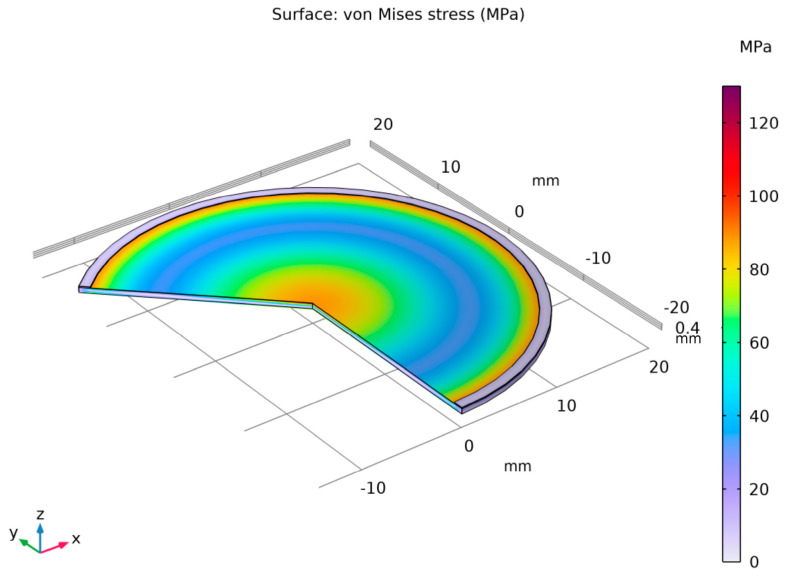
Distribution of von Mises stresses within the working plane, accompanied by the visible deformation.

**Figure 14:**
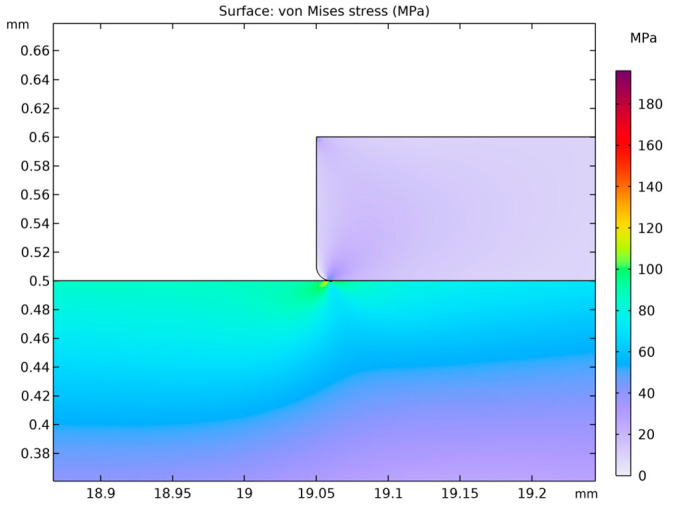
Local region of maximum stresses in a thin steel plate.

A thin plate made of 316 stainless steel behaves in accordance with the elastic model, which means that when subjected to pressure loads, it deforms reversibly without exceeding the material’s yield strength. The yield limit for 316 stainless steel is known, and the results of the analysis (Figure 12, Figure 13 and Figure 14) indicate that the maximum von Mises stresses obtained in the membrane do not exceed this value. This confirms that the material remains within the elastic region and no permanent deformations of the structure occur.

A thin plate made of 316 stainless steel behaves according to the elastic model, meaning that when subjected to pressure loads, it deforms reversibly without exceeding the material’s yield strength. This limit for 316 stainless steel is $2.80\times 10^{8}\;Pa$, and the analysis results (Figure 12, Figure 13 and Figure 14) indicate that the maximum von Mises stresses obtained in the membrane do not exceed $1.21\times 10^{8}\;Pa$. This confirms that the material remains within the elastic region and that no permanent deformation of the structure occurs. The pressure difference between the external side, equal to ${1.013\times 10}^{5}$ Pa, and the very low internal pressure of ${1\times 10}^{-4}$ Pa induce both tensile and compressive stresses in different parts of the membrane. These stresses generate in-plane deformations, while simultaneously causing a deflection of the membrane outward, manifested as maximum displacements on the order of $1.34\times 10^{-4}\;m$. Such deformations remain within the physical elasticity range of the material, ensuring that the membrane returns to its original shape once the load is removed.

**Figure 15:**
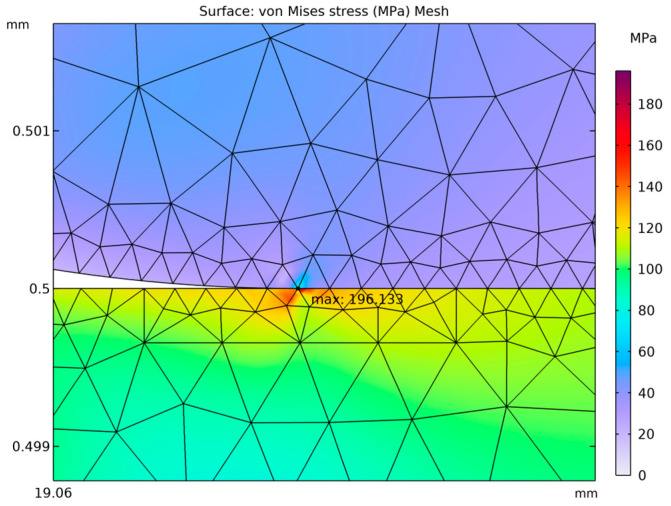
Region of maximum stress in the thin steel plate.

The greatest deformation values, like those observed in the model with the corrugated metal plate, which are crucial for the elastic behavior of the thin steel sheet, were identified in the region corresponding to the adhesive–steel sheet interface. Locally, the stresses in this area reach values up to $2.0\times 10^{8}\;Pa$ as shown in Figure 15. The magnitudes attained in this region remain within the elastic strain range of the material.

The elastic behavior of the plate ensures that the entire structure exhibits resistance to repetitive pressure variations, which is of critical importance in vacuum pressure measurement applications. Elastic deformations minimize material fatigue and reduce the likelihood of microcrack formation, thereby extending the device’s operational lifespan without failure.

## 4. Conclusions

The thickness of the membrane is crucial for sensor design, as it directly affects its sensitivity, measuring range, and mechanical and thermal resistance. The thinner the membrane, the greater its deformation under the influence of the measured pressure or force, which increases the sensitivity of the sensor, but, at the same time, reduces its resistance to damage and the maximum pressure range that it can safely measure. A thin membrane makes it easier to detect even small changes in pressure or force, which is beneficial in applications requiring high sensitivity. But it should be noted that a thicker membrane provides greater mechanical strength and allows for the measurement of larger pressure ranges, which is an essential element in this document due to the wide measurement range that the sensor should offer, determined on the basis of the typical pressure inside the SN vacuum extinguishing chamber, which is in the order of 10^−3^ Pa. A membrane that is too thin may lead to damage, while one that is too thick may limit the measurement range or reduce the response dynamics of the sensor. Therefore, a key element in the design of the sensor head with a membrane is the precise determination of its thickness to minimize the risk of plastic deformation and damage to the component during normal operation.

The developed pressure measurement system uses a 316 L steel membrane with parameters E = 2 × 10^11^ Pa, ν = 0.28, ρ = 7980 kg/m^3^, and yield strength 2.8 × 10^8^ Pa, with an outer diameter of 2.54 × 10^−2^ m, an inner diameter of 2.22 × 10^−2^ m, and a thickness of t = 5.08 × 10^−5^ m, ensuring operation in a fully elastic range for pressure differences from Δp = 7 × 10^−4^ Pa (inner side) to 1.013 × 10^5^ Pa (outer side), with maximum von Mises stress not exceeding 1.96 × 10^8^ Pa. The central deflection of the membrane, described by an analytical relationship, allows for a sensitivity of 9.1 × 10^−10^ m/Pa for the variant with a diameter of 3.81 × 10^−2^ m (active area A = 1.140 × 10^−3^ m^2^), which corresponds to an increase of approximately 225% compared to the variant with a diameter of 2.54 × 10^−2^ m (A = 5.067 × 10^−4^ m^2^). Integration with an FBG fiber-optic sensor (Lublin University of Technology, Lublin, Poland), attached with epoxy adhesive (E = 5 × 10^9^ Pa, ν = 0.35, tensile strength 4.2 × 10^10^ Pa), enables precise detection of the Bragg wavelength shift $\lambda_{B}$ with a resolution of the order of picometres, which creates conditions for reliable monitoring of the vacuum condition in the extinguishing chambers of medium-voltage devices.

Experimental tests conducted on corrugated membranes with a thickness of t = 2.0 × 10^−5^ m revealed significant deviations from the characteristics declared by the manufacturer (including a deflection of 2.0 × 10^−5^ m at a pressure of 6.89 × 10^2^ Pa for 316 steel), resulting largely from the heterogeneity of the adhesive layer and the lack of coaxiality, which cause hysteresis and nonlinearity of the characteristics. Numerical calculations using the finite element method indicate the occurrence of critical stress concentrations in the membrane–adhesive–body contact area (σ_vM exceeding 2.8 × 10^8^ Pa in the corrugated design), which necessitates the use of a membrane with a smooth geometry, the introduction of a rounded adhesive layer edge with a radius of R = 1.0 × 10^−4^ m, and high-precision assembly to achieve the required repeatability. The optimized configuration meets the criteria for integration with medium-voltage equipment but imposes limitations on miniaturization due to the need to maintain a minimum membrane thickness of t ≥ 50 × 10^−6^ m.

It must be emphasized that one of the key diagnostic methods for vacuum devices is the identification of internal pressure levels. Therefore, given the excellent insulating properties of vacuum at specific pressure conditions, real-time pressure measurement remains a critical factor. For this reason, the development of a reliable and durable sensor is essential for the advancement of this sector of industry.

## Figures and Tables

**Table 1:** Declared technical parameters of the membranes used [20].

LP	Pressure [Pa]	Membrane Deflection [mm]	
		Stainless Steel	INCOTEL 718
1	$6.89\times 10^{0}$	$2.00\;\times \;10^{-5}$	$9.00\times 10^{-5}$
2	$6.89\times 10^{1}$	$5.00\times 10^{-5}$	$1.00\times 10^{-4}$
3	$6.89\times 10^{2}$	$3.00\times 10^{-4}$	$3.00\times 10^{-4}$
4	$2.75\times 10^{3}$	$1.30\times 10^{-3}$	$1.20\times 10^{-3}$
5	$4.26\times 10^{3}$	$2.30\times 10^{-3}$	$2.20\times 10^{-3}$
6	$6.89\times 10^{3}$	$3.30\times 10^{-3}$	$3.10\times 10^{-3}$
7	$3.44\times 10^{4}$	$1.60\times 10^{-2}$	$1.40\times 10^{-2}$

**Table 2:** Technical parameters of the membrane subjected to simulation analysis.

LP	Parameter	Value
1	Material	316 stainless steel
2	Pressure inside the system	$7\times 10^{-4}\;Pa$
3	Pressure outside the system	$1.013\times 10^{5}\;Pa$
4	Outer diameter of the membrane	$2.54\times 10^{-2}\;mm$
5	Internal diameter of the membrane	$2.2225\times 10^{-2}\;m$
6	Membrane thickness	$5.08\times 10^{-5}\;m$
7	Membrane yield strength	$2.8\times 10^{8}\;Pa$
8	Young’s modulus for the membrane	$2\times 10^{11}\;Pa$
9	Poisson Ratio	0.28
10	Density	7980 kg m^−3^

**Table 3:** Technical parameters of the adhesive used.

LP	Parameter	Value
1	Thermal conductivity	1.2–1.22 W/(m × K)
2	Tensile strength	42 MPa (42 N/mm^2^)
3	Dielectric strength	8.5 kV/mm
4	Breakdown voltage	19.8 kV
5	Hardness	68
6	Density after mixing	$2110\;kg\times m^{-3}$
7	Resistivity	$1\times 10^{12}\Omega \times cm$
8	Operating temperature	od −40 °C do +150 °C
9	Glass transition temperature (Tg)	9 °C
10	Coefficient of thermal expansion (CTE)	before Tg: 47 ppm/°C after Tg: 164 ppm/°C
11	Young’s modulus	$5\times 10^{9}\;Pa$
12	Poisson Ratio	0.35

**Table 4:** Comparative analysis of the maximum deformation for membranes with diameters Ø 2.54 × 10^−2^ m and Ø 3.81 × 10^−2^ m.

$Membrane\;\emptyset =2.54\times 10^{-2}\;m$	$Membrane\;\emptyset =3.81\times 10^{-2}\;m$
$y_{max}=\frac{{3\left(1-\vartheta^{2}\right)Pr}^{4}}{16{Et}^{3}}$	$y_{max}=\frac{{3\left(1-\vartheta^{2}\right)Pr}^{4}}{16{Et}^{3}}$
$y_{max}=\frac{{3\times \left(1-0.28^{2}\right)\times {(1.013\times 10}^{3})\times (1.27\times 10^{-2})}^{4}}{16\times {(2\times 10}^{11})\times {{(1\times 10}^{-5})}^{3}}$	$y_{max}=\frac{{3\times \left(1-0.28^{2}\right)\times {(1.013\times 10}^{3})\times (1.905\times 10^{-2})}^{4}}{16{\times (2\times 10}^{11})\times {{(1\times 10}^{-5})}^{3}}$
$y_{max}=22.48$	$y_{max}=113.79$
$\frac{113.79}{22.48}=5.06$	

**Table 5:** Analysis of the linear behavior of a metal plate.

LP	P	Sensitivity	Deflection	Deflection
	[Pa]	[m/Pa]	[m]	[nm]
1	$1.0\times 10^{-4}$	$9.103\times 10^{-10}$	$9.103\times 10^{-14}$	$9.10\times 10^{-5}$
2	$5.0\times 10^{-4}$		$9.103\times 10^{-13}$	$4.55\times 10^{-4}$
3	$1.0\times 10^{-3}$		$9.103\times 10^{-13}$	$9.10\times 10^{-4}$
4	$5.0\times 10^{-3}$		$9.103\times 10^{-12}$	$4.55\times 10^{-3}$
5	$1.0\times 10^{-2}$		$9.103\times 10^{-12}$	$9.10\times 10^{-3}$
6	$5.0\times 10^{-2}$		$9.103\times 10^{-11}$	$4.55\times 10^{-2}$
7	$1.0\times 10^{-1}$		$9.103\times 10^{-11}$	$9.10\times 10^{-2}$
8	$5.0\times 10^{-1}$		$9.103\times 10^{-10}$	$4.55\times 10^{-1}$
9	$1.0\times 10^{0}$		$9.103\times 10^{-10}$	$9.10\times 10^{-1}$
10	$5.0\times 10^{0}$		$9.103\times 10^{-9}$	$4.55\times 10^{0}$
11	$1.0\times 10^{1}$		$9.103\times 10^{-9}$	$9.10\times 10^{0}$
12	$5.0\times 10^{1}$		$9.103\times 10^{-8}$	$4.55\times 10^{1}$
13	$1.0\times 10^{2}$		$9.103\times 10^{-8}$	$9.10\times 10^{1}$
14	$5.0\times 10^{2}$		$9.103\times 10^{-7}$	$4.55\times 10^{2}$
15	$1.0\times 10^{3}$		$9.103\times 10^{-7}$	$9.10\times 10^{2}$
16	$5.0\times 10^{3}$		$9.103\times 10^{-6}$	$4.55\times 10^{3}$
17	$1.0\times 10^{4}$		$9.103\times 10^{-6}$	$9.10\times 10^{3}$
18	$5.0\times 10^{4}$		$9.103\times 10^{-5}$	$4.55\times 10^{4}$
19	$1.0\times 10^{5}$		$9.103\times 10^{-5}$	$9.10\times 10^{4}$

## Data Availability

The original contributions presented in this study are included in the article. Further inquiries can be directed to the corresponding author.

## References

[1] Li Q., Xu F., Lai X., Chen X. Research on Intelligent Integrated Detection System with Fiber Optic Imaging in High Voltage Switchgear Environment. Proceedings of the 2024 IEEE 6th International Conference on Civil Aviation Safety and Information Technology (ICCASIT).

[2] Dong C., Wei B., Cao S., Liu Q., Dai X. Research on On-Line Monitoring Technology of Circuit Breakers Based on Multi-Sensor Information Data Fusion Technology. Proceedings of the 2023 International Conference on Power System Technology (PowerCon).

[3] Zhang F., Wu F., Yuan H., Yang A., Wang X., Rong M. Online Monitoring Technology for Vacuum Degree of Vacuum Circuit Breakers Based on Fiber-Optical Laser-Induced Breakdown Spectroscopy. Proceedings of the 2024 3rd International Conference on Power Systems and Electrical Technology (PSET).

[4] Cruz Feliciano A.J., Jin Z., Graber L. (2025). Supercritical Fluids as Alternative Insulation and Arc-Quenching Medium. Appl. Sci..

[5] Nicollini G., Devecchi D. (2018). MEMS Capacitive Microphones: Acoustical, Electrical, and Hidden Thermal-Related Issues. IEEE Sens. J..

[6] Sant L., Fuldner M., Bach E., Conzatti F., Caspani A., Gaggl R., Baschirotto A., Wiesbauer A. (2022). A 130dB SPL 72dB SNR MEMS Microphone Using a Sealed-Dual Membrane Transducer and a Power-Scaling Read-Out ASIC. IEEE Sens. J..

[7] Le Roux A.-L., Quiroga X., Walani N., Arroyo M., Roca-Cusachs P. (2019). The plasma membrane as a mechanochemical transducer. Philos. Trans. R. Soc. Lond. B Biol. Sci..

[8] Belwanshi V., Rane K., Kumar V., Pramanick B. (2023). Design Guidelines for Thin Diaphragm-Based Microsystems through Comprehensive Numerical and Analytical Studies. Micromachines.

[9] Hall N.A., Okandan M., Littrell R., Bicen B., Degertekin F.L. (2008). Simulation of Thin-Film Damping and Thermal Mechanical Noise Spectra for Advanced Micromachined Microphone Structures. J. Microelectromech. Syst..

[10] Liang M., Fang X., Wu G., Xue G., Li H. (2017). A fiber bragg grating pressure sensor with temperature compensation based on diaphragm-cantilever structure. Optik.

[11] Pachava V.R., Kamineni S., Madhuvarasu S.S., Putha K. (2014). A high sensitive FBG pressure sensor using thin metal diaphragm. J. Opt..

[12] Huang J., Zhou Z., Zhang D., Wei Q. (2013). A Fiber Bragg Grating Pressure Sensor and Its Application to Pipeline Leakage Detection. Sage J..

[13] Rajita G., Banerjee D., Mandal N., Bera S.C. (2015). Design and Analysis of Hall Effect Probe-Based Pressure Transmitter Using Bellows as Sensor. IEEE Trans. Instrum. Meas..

[14] Zhao L.B., Fang X.D., Zhao Y.L., De Jiang Z., Li Y. (2011). A High Pressure Sensor with Circular Diaphragm Based on MEMS Technology. Key Eng. Mater..

[15] Rosca A., Rosca D., Nastasescu V. Consideration on Stainless Steel Plate Plastic Deformed by Fluid Impact Substances and Pressured Nitrogen Blasting. January 2008; Volume 3. http://www.ucv.ro.

[16] Jindal S.K., Sethi K., Patel I., Kumar A., Raghuwanshi S.K. (2021). A Semi-Analytical and Computationally Efficient Method to Calculate the Touch-Point Pressure and Pull-In Voltage of a MEMS Pressure Sensor with a Circular Diaphragm. IEEE Sens. J..

[17] Fan Q., Jia Z., Feng D., Yong Z. (2021). Highly sensitive FBG pressure sensor based on square diaphragm. Optik.

[18] Węgierek P., Kostyła D., Lech M. (2023). Directions of Development of Diagnostic Methods of Vacuum Medium-Voltage Switchgear. Energies.

[19] Chmielak W. (2014). Review of methods of diagnostics of the vacuum in vacuum circuit breakers. Przegląd Elektrotechniczny.

[20] Węgierek P., Kostyła D., Lech M., Kozak C., Zielonka A. (2023). Pressure Monitoring in Medium-Voltage Vacuum Interrupters. Energies.

[21] Enakoutsa K., Li Y. (2025). Metal plasticity and nonlocal damage modeling: A thermodynamically consistent GSM approach with computational implications. Math. Mech. Solids.

[22] Zeng G., Huang Z., Deng B., Ge R. (2025). Crystal Plasticity Finite Element Simulation of Tensile Fracture of 316L Stainless Steel Produced by Selective Laser Melting. Metals.

[23] Dhoriyani U., Goswami R.D., Kanekal D., Jindal S.K., Tiwari R. Analysis of MEMS Piezoresistive Pressure Sensor with Dual-Island Structures using Circular Silicon Diaphragm. Proceedings of the 2024 Global Conference on Communications and Information Technologies (GCCIT).

[24] Koch J., Brinkmann L.M., Kassner A., Dencker F., Wurz M.C. Silicon-based membrane pressure sensor for inline monitoring of pressure and hermeticity of small-volume bonded packages. Proceedings of the 2024 IEEE 74th Electronic Components and Technology Conference (ECTC).

[25] Lian Y.-S., Sun J.-Y., Zhao Z.-H., He X.-T., Zheng Z.-L. (2020). A Revisit of the Boundary Value Problem for Föppl–Hencky Membranes: Improvement of Geometric Equations. Mathematics.

[26] Dagamseh A., Al-Bataineh Q., Al-Bataineh Z., Daoud N.S., Alsaad A., Omari A. (2020). Modeling of a square-shape ZnO, ZnS and AlN membrane for mems capacitive pressure-sensor applications. Int. J. Simul. Multidiscip. Des. Optim..

[27] Vamshi N., Deepak A. (2022). Simulation of Circular Shaped SiC Diaphragm based Pressure Sensor and Comparing Its Deflection Characteristics with Square Shaped Diaphragm for Improved Sensor Performance. J. Pharm. Negat. Results.

[28] Das K., Dutta H.S. (2024). Improved Sensitivity of MEMS-based Piezoresistive Pressure Sensor using Silicon Nitride Diaphragm. J. Integr. Circuits Syst..

[29] Orthner M.P., Buetefisch S., Magda J., Rieth L.W., Solzbacher F. (2010). Development, fabrication, and characterization of hydrogel based piezoresistive pressure sensors with perforated diaphragms. Sens. Actuators A Phys..

[30] Mott P.H., Roland C.M. (2013). Limits to Poisson’s ratio in isotropic materials-general result for arbitrary deformation. Phys. Scr..

[31] Østergaard M.B., Hansen S.R., Januchta K., To T., Rzoska S.J., Bockowski M., Bauchy M., Smedskjaer M.M. (2019). Revisiting the Dependence of Poisson’s Ratio on Liquid Fragility and Atomic Packing Density in Oxide Glasses. Materials.

[32] Inui M., Huang Y., Onozuka H., Umezu N. (2020). Geometric simulation of power skiving of internal gear using solid model with triple-dexel representation. Procedia Manuf..

[33] Pernu T., Saarilahti J., Kyynarainen J., Sillanpaa T. (2023). Ultra-High Sensitivity Surface-Micromachined Capacitive Differential Pressure Sensor for Low-Pressure Applications. J. Microelectromech. Syst..

